# SPONTANEOUS MUTATION ACCUMULATION IN MULTIPLE STRAINS OF THE GREEN ALGA, *CHLAMYDOMONAS REINHARDTII*

**DOI:** 10.1111/evo.12448

**Published:** 2014-07-09

**Authors:** Andrew D Morgan, Rob W Ness, Peter D Keightley, Nick Colegrave

**Affiliations:** 1 Institute of Evolutionary Biology, School of Biological Sciences, University of EdinburghKing's Buildings, Edinburgh, EH9 3JT, United Kingdom

**Keywords:** Genomic deleterious mutation rate, mutation accumulation, mutational effect, spontaneous mutation

## Abstract

Estimates of mutational parameters, such as the average fitness effect of a new mutation and the rate at which new genetic variation for fitness is created by mutation, are important for the understanding of many biological processes. However, the causes of interspecific variation in mutational parameters and the extent to which they vary within species remain largely unknown. We maintained multiple strains of the unicellular eukaryote *Chlamydomonas reinhardtii*, for approximately 1000 generations under relaxed selection by transferring a single cell every ∼10 generations. Mean fitness of the lines tended to decline with generations of mutation accumulation whereas mutational variance increased. We did not find any evidence for differences among strains in any of the mutational parameters estimated. The overall change in mean fitness per cell division and rate of input of mutational variance per cell division were more similar to values observed in multicellular organisms than to those in other single-celled microbes. However, after taking into account differences in genome size among species, estimates from multicellular organisms and microbes, including our new estimates from *C. reinhardtii*, become substantially more similar. Thus, we suggest that variation in genome size is an important determinant of interspecific variation in mutational parameters.

Spontaneous mutation is ultimately the source of all genetic variation. Consequently, mutation is central to many aspects of biology, including the genetic basis of heritable disease, biodiversity conservation, agricultural breeding, population genetics, and evolution. A comprehensive description of the process of mutation and the effects of mutant alleles on the phenotype is therefore fundamental to our understanding of biological processes. For example, the rate at which a species can adapt to a novel or changing environment is largely determined in the short term by the amount of genetic variation maintained in a population and in the longer term by the availability of new beneficial mutations. However, it is widely held that most new spontaneous mutations that affect fitness are harmful (Keightley and Lynch 2003; Loewe and Hill 2010), and can have a substantial cumulative effect on the fitness of populations (Charlesworth and Charlesworth 1998; Keightley and Eyre-Walker 1999; Lynch et al. 1999). This can lead, for example, to the maintenance of sex and recombination, driven by the increased capacity of sexual populations to efficiently purge deleterious alleles (Kondrashov 1988; Otto 2009; Hartfield and Keightley 2012).

New spontaneous mutations are undoubtedly important in many evolutionary processes, but the fitness effects of naturally occurring new mutations have been difficult to study. Although many new mutations are expected to occur per generation in a population, each one tends to be present at a low frequency (1/*N* when it originally arises, where *N* is the number of gene copies in the population). The vast majority of alleles found segregating at detectable frequencies in natural populations have already been subject to selection and are likely to have small fitness effects. As a result, population genetic approaches to infer the distribution of mutational effects are therefore most effective at estimating the effects of mildly beneficial or weakly deleterious mutations (Keightley and Eyre-Walker 2010).

A common approach to overcome this challenge is to allow mutations to build up in the near absence of natural selection in a mutation accumulation (MA) experiment in the laboratory. MA reduces the effectiveness of selection by bottlenecking experimental populations, making transmission to the next generation random with respect to fitness, allowing all but the most strongly deleterious mutations to accumulate. The change in fitness among replicate MA lines compared to controls can then be used to estimate the genomic rate and the distribution of effects of new mutations (for a review, see Halligan and Keightley 2009).

Typically, fitness declines and among-line variance in fitness increases in MA experiments, but the magnitude of these changes varies greatly among species (Baer et al. 2007). In particular, there appear to be substantial differences between unicellular and multicellular organisms. The multicellular species that have been studied to date (*Amsinckia douglasiana* [Schoen 2005 ], *A. gloriosa* [Schoen 2005 ], *Arabidopsis thaliana* [Schultz et al. 1999; Shaw et al. 2000 ], *Caenorhabditis elegans* [Keightley and Caballero 1997; Baer et al. 2005 ], *C. briggsae* [Baer et al. 2005 ], *Drosophila melanogaster* [García-Dorado et al. 1998; Charlesworth et al. 2004 ], *Oscheius myriophilia* [Baer et al. 2005 ], and *Daphnia pulicaria* [Schaack et al. 2013 ]) show both a greater decline in fitness and greater increase in genetic variance for fitness per generation than the two unicellular organisms that have been studied (*Saccharomyces cerevisiae* [Zeyl and DeVisser 2001; Joseph and Hall 2004; Dickinson 2008 ] or *Escherichia coli* [Kibota and Lynch 1996 ]). This difference remains even if fitness change is estimated per cell division rather than per generation, and so is not simply a consequence of the fact that the germline of a multicellular eukaryotes typically goes through multiple divisions each generation.

Variation among species in the outcome of MA may reflect fundamental differences in the genetics of single-celled microbes compared to multicellular eukaryotes. Single-celled microbes generally have lower mutation rates per base pair, which may be related to their larger effective population sizes, allowing selection to remove mutator alleles more efficiently from a population (Lynch 2010). However, microbes and multicellular organisms have similar mutation rates per base pair per cell division (Drake et al. 1998). A relevant factor is that microbes have many genes (accessory genes) that are expressed only in specific environmental conditions. The number of accessory genes in bacteria is believed to exceed the number of genes required for survival in minimal media (Koonin 2003), and in yeast ∼60% of open reading frames can be knocked out without producing a measurable effect of fitness in either rich or minimal media (Winzeler et al. 1999). The abundance of sites in the genome where mutant alleles are conditionally neutral in a particular environment may partly explain why MA experiments in single-celled microbes have tended to show smaller changes in fitness than multicellular organisms.

Genome size may also play a role in the outcome of an MA experiment. All else being equal, a species with a larger genome will accumulate more mutant alleles than a species with a smaller genome, and might therefore show a greater decline in fitness. A refinement of this idea is the concept of the “effective genome size,” which is the product of genome size and the fraction of the genome that is functional (Drake et al. 1998). For example, the outcome of MA may depend more strongly on the number of protein coding sites in the genome than overall genome size, if the genome has a large number of nonfunctional sites. Because the genomes of *S*. *cerevisiae* (12.1 Mbp; Goffeau et al. 1996) and *E*. *coli* (4.6–5.7 Mbp; Lukjancenko et al. 2010) are substantially smaller than those of *A. thaliana* (157 Mbp; The Arabadopsis Genome Initiative 2000), *D*. *melanogaster* (140 Mbp; Adams et al. 2000), and *C*. *elegans* (97 Mbp; The *C. elegans* Sequencing Consortium 1998), variation in effective genome size may partly explain differences in the responses to MA in these species.

In addition to variation in mutational parameters among species, there is the possibility of variation within a species. However, few MA studies have included multiple genotypes of the same species to address this question directly. Lower bound estimates of the genome-wide deleterious mutation rate (*U*) obtained using the Bateman–Mukai (BM) method (Bateman 1959; Mukai 1964) vary substantially between different MA experiments in *D. melanogaster* (Mukai 1964; Mukai et al. 1972; Fernandez and Lopez-Fanjul 1996; García-Dorado et al. 1998; Fry et al. 1999) and estimates of the rate of decline of variation in fitness differed significantly among strains of *D. pulicaria* (Schaack et al. 2013). However, there is only one study that we are aware of that compared strains of the same species within a single experiment to study variation in *U* (Baer et al. 2005). In that study, there was significant variation in estimates of *U* and in the rate of change of fitness between two genotypes in two species of nematodes. For a number of reasons, we might expect to find variation in the outcome of MA within a species. First, there is limited but clear evidence for mutation rate variation within species. In bacteria, for example, mutator genotypes that have mutation rates as much as 100-fold higher than wild-type strains have been isolated from natural populations (Matic et al. 1997; Sundin and Weigand 2007). In *Chlamydomonas reinhardtii*, sequencing of the genomes of MA lines initiated from two different strains yielded estimates of the per nucleotide site mutation rate that differ fivefold (Ness et al. 2012; Sung et al. 2012). In *D. melanogaster*, there is also evidence for variation in the mutation rate per nucleotide site per generation between strains (Keightley et al. 2009, 2014; Schrider et al. 2013). Furthermore, there is evidence from *Drosophila* that individuals carrying deleterious alleles have elevated mutation rates (Sharp and Agrawal 2012). Such an effect would lead to a greater reduction in fitness as a consequence of MA in initially less-fit genotypes, a pattern that has been observed in nematodes (Baer et al. 2005). Another possibility is that the underlying distribution of fitness effects may differ among individuals or may depend on genetic background (Barrick et al. 2010; Sousa et al. 2012). Although the majority of mutations are expected to be deleterious, the fraction of beneficial mutations may vary. For example, a higher proportion of mutations are expected to be beneficial in poorly adapted genotypes than in better adapted genotypes (Fisher 1930).

In this study, we conduct MA in multiple, genetically diverse strains of *C. reinhardtii*. This unicellular microbe has a large effective population size (Ness et al. 2012) and shares characteristics with microbial model species, such as *S*. *cerevisiae* and *E. coli* and multicellular eukaryote model species such as *A*. *thaliana*, *C*. *elegans*, and *D*. *melanogaster*. Previous studies suggest that its mutation rate per base pair per generation is similar to *S*. *cerevisiae* and much lower than other multicellular eukaryotes (Ness et al. 2012; Sung et al. 2012). However, unlike other model single-cell microbes, including *E. coli* and *S. cerevisiae*, the genome of *C*. *reinhardtii* is relatively large (112 Mbp; Merchant et al. 2007), and similar to that of *A*. *thaliana*, *C*. *elegans*, and *D*. *melanogaster*. It has a large and complex proteome (∼13 × 10^6^ codons) that is substantially larger than *S. cerevisiae*, *E. coli*, *C. elegans*, and *D. melanogaster*, and is similar to *A*. *thaliana* (Massey 2008). We allow mutations to accumulate in mitotically dividing haploid cells, and assay the effects of mutations in the hemizygous state to address the following questions: (1) To what extent does the accumulation of spontaneous mutations affect the mean fitness of *C*. *reinhardtii* populations and variation in fitness among replicate populations? (2) Is there evidence for within species variation in the response to MA? (3) How does the effect of MA on fitness in *C. reinhardtii* compare with the effects observed in other unicellular and multicellular species?

## Methods

### STRAINS

We allowed spontaneous mutations to accumulate in six wild strains of *C. reinhardtii* obtained from the Chlamydomonas Resource Center. The strains were chosen to broadly cover the geographic range of the species, and previous work suggests that they are genetically diverse (Smith and Lee 2008; Table1). Three of the strains were of the MT+ mating type and three were of the MT– mating type. To confirm the identities of the strains, we sequenced the *ypt4*-VI and *ypt4*-VII introns (Liss et al. 1997). Each of our sequences matched the published sequences.

**Table 1:** Strains used in the MA experiment

Strain	Isolated (location/year)	Mating type
CC-1373	South Deerfield, MA, USA/1945	+
CC-1952	MN, USA/1986	−
CC-2342	PA, USA/1989	−
CC-2344	PA, USA/1989	+
CC-2931	Durham, NC, USA/1991	−
CC-2937	QC, Canada/1993	+

### CULTURE CONDITIONS

Algal strains were grown in Bold's liquid medium or on Bold's agar plates, the standard media for culture of *C. reinhardtii* in the laboratory. Bold's medium is a simple salt medium containing only essential minerals. *Chlamydomonas reinhardtii* is photosynthetic and requires no additional nutrients for growth. All cultures were grown at 25°C under white light.

### MUTATION ACCUMULATION

To initiate the MA lines, each of the six strains was grown for four days in a separate 30-ml universal tube containing 10 ml Bold's medium, shaken, with loose caps to allow gaseous exchange. To preserve the ancestral strains of the MA lines, several aliquots from each of these cultures were frozen in liquid nitrogen, as detailed below.

Aliquots of the cultures were streaked onto separate Bold's agar plates and allowed to grow for four days to produce individual colonies. From each of the six strains, we randomly selected 15 individual colonies and used these to initiate 15 MA lines (for a total of 90 MA lines). Each colony was streaked onto a fresh agar plate by initially placing the colony in a 10 μl spot of Bold's medium to allow separation of the cells and ensure that the cells would form new individual colonies.

To allow mutations to accumulate, we bottlenecked the MA lines at regular intervals to a single cell. At each transfer, a colony originating from a single cell was selected using a random coordinate system and streaked onto a fresh agar plate. Initially, the period between transfers alternated between three and four days. This regime was chosen to minimize the amount of growth within a colony to reduce competition between newly arising mutants, while allowing a sufficient period of growth for colonies to become visible even if they had accumulated mutations that substantially reduced their growth rate. Previous estimates of the number of divisions on plates of one of our ancestral strains (CC-2937) showed that the strain could achieve more than eight divisions in a three-day period of growth (Ness et al. 2012), which is substantially more than the six divisions required for a colony to be visible at transfer. Thus, even mutations with substantial effects on fitness should accumulate under this protocol.

We regularly performed tests to determine whether all of the colonies on a plate had become visible at the time of transfer. After 19 transfers, it was determined that a small minority of colonies (∼3%) could not be seen after three days in several lines, so we changed to a 4–5–5-day transfer cycle for the duration of the experiment, up to a total of 85 transfers. We continued to test regularly whether additional colonies became visible after the transfer period.

We retained the plates from the three prior transfers as backups at room temperature in dim light. In the event of a transfer failing, and no colonies growing on a plate, the transfer was repeated from the most recent backup plate. About two-thirds of the MA lines experienced this kind of failed transfer at least once. Occasionally, an MA line failed to transfer repeatedly, in which case the transfer was attempted using an earlier backup plate. If a line failed to transfer three times or a line became contaminated with bacteria or fungus, we restarted the line using the recent stock that had been previously cryopreserved in liquid nitrogen every 15 transfers. We needed to rejuvenate MA lines from their frozen stocks in only two cases in the whole experiment.

### FREEZING AND RECONDITIONING OF CULTURES

We froze samples from the MA lines regularly at intervals of approximately 15 transfers (∼150 generations) using a modified version of a previously published protocol (Crutchfield et al. 1999). For each MA line, we minimized the chance of picking a colony containing a single novel genotype by freezing five-day-old high-density liquid cultures derived from several colonies. We also froze the ancestral strains using samples of the original cultures that had each been used to initiate the MA lines for each strain (see above). For freezing, we mixed 0.9 ml of each dense culture with 0.9 ml of cryopreservation medium, comprising 10% methanol v/v in Bold's medium. The mixtures were then placed at –80°C in a container containing isopropanol that allowed controlled cooling at a rate of ∼1°C/min. After 4 h, the tubes were cryopreserved in liquid nitrogen.

To recondition the frozen cultures for growth, cultures were thawed in a water bath at 35°C for 5 min. The entire contents of a tube were then placed in a 30-ml universal tube containing 10 ml of Bold's medium with loose caps to allow gaseous exchange and incubated at 25°C under white light for seven days.

### ESTIMATING NUMBERS OF GENERATIONS DURING MA

We estimated the number of generations undergone by each MA line over the course of the experiment by measuring the number of cells in colonies grown on agar plates after a period of growth equivalent to the times between transfers in the experiment. *Chlamydomonas reinhardtii* divides by binary fission, so the number of generations in a specified time period can be calculated from the number of cells within a colony.

To estimate the number of generations, we reconditioned the MA lines and their ancestral strains. These reconditioned cultures were then serially diluted, plated onto Bold's agar plates and grown for three, four, or five days, that is, the lengths of time between transfers in the experiment. For each MA line or ancestral strain, we then selected one plate containing between 10 and 200 colonies from its dilution series. The number of colonies was counted, and then the plate was flooded with Bold's medium and left for 2 h to allow the cells to disperse into the liquid medium. We then removed the liquid medium containing the cells and recorded its volume. *Chlamydomonas reinhardtii* has a minimum generation time of 6 h under perfect growing conditions, so the number of cells should not have significantly increased in the 2-h period.

To determine the total number of cells on the plate, we used a Casy Counter to determine the density of cells within the suspension removed from the plate. The average number of cells per colony (*N_f_*) was then estimated as the number of cells removed from the plate divided by the number of colonies on the plate. We then calculated the number of generations (*G*) for each transfer period using the following equation:

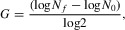
1where *N*_0_ = 1 is the initial number of cells in a colony, because each colony was founded by a single cell. We repeated the whole assay, so that we had two replicates measures for each ancestral strain and MA line, and took an average to get a single estimate.

To estimate the total number of generations of MA undergone by each MA line, we multiplied the number of generations achieved in three, four, or five days for that line by the number of times that this transfer period occurred during that line's MA. To attempt to account for any change of mean generation time during the experiment, we determined the total number of transfers undergone by each line at the end of the experiment, and assumed that each line grew at the same rate as its ancestral strain up to its middle transfer, and thereafter at the same rate as estimated for that line at the end of the experiment.

### FITNESS ASSAYS

To estimate the fitness of the MA lines and their ancestors, we estimated their maximal growth rate (μ_max_) in Bold's liquid medium as described below. MA line cultures were first reconditioned after freezing as described above, then inoculated into 30-ml universal tubes containing 5 ml of Bold's medium. At this point, we also inoculated each ancestral strain into nine separate universal tubes, to create nine pseudolines per ancestral strain. These pseudolines enabled us to estimate the among-line environmental variance and to distinguish environmental from mutational effects.

After three days of growth in the universal tubes, we created five replicate cultures for each of their nine pseudoline control lines of each strain and five replicate cultures for each of the 15 MA lines of each strain. These replicate cultures were produced by inoculating the cultures from the universal tubes into individual wells of 48-well plates containing 1.5 ml of Bold's medium and allowing them to grow for four days. This further four days of conditioning, along with the conditioning in universal tubes, was intended to minimize any effects of freezing on growth during the fitness assay. The cultures were distributed at random across three separate blocks, each block consisting of five separate 48-well plates (for a total of 15 plates). Within each block, there were three ancestral pseudolines and five MA lines from each of the original strains.

To estimate μ_max_ for each replicate of each line, we measured absorbance through time in 96-well plates in a microplate reader. A total of 2 μl of each culture was transferred from a 48-well plate into the corresponding central 48 wells of a 96-well plate. Each well of the 96-well plates contained 200 μl of Bold's medium. The outer wells were also filled with media to slow evaporation and humidify the central wells. Plates were placed at 25°C under white light for seven days. We measured the absorbance at 650 nm twice daily in the first fitness assay and three times daily in the second assay over a seven-day period.

To estimate fitness for each replicate, we used the nlstools package in R to fit a growth curve to the absorbance data. Growth was modeled using a Baranyi model without lag phase, which provides an estimate of the maximum rate of growth (μ_max_) (Baranyi and Roberts 1994).

### STATISTICAL ANALYSIS

The analysis was carried out using R version 3.0.0 (R Core Team 2013). In the first run of the fitness assay, the ancestral pseudolines for strain CC-2937 became contaminated with bacteria. Initial data exploration showed that the fitness estimates for these control lines were anomalous, being significantly lower than any other lines in the assay, including the MA lines derived from them. They were also significantly lower than the estimates of the same control lines in the second run of the assay. We therefore analyzed the data from the two runs of the fitness assay separately, excluding data from CC-2937 from the analyses of the first fitness assay.

We use a general linear mixed model to obtain REML estimates of the among-line and within-line variance components for the fitness of the 15 MA lines derived from each ancestral strain and for the 9 ancestral pseudolines. The model includes the five replicate measures of fitness (maximal growth, μ_max_) for each MA line as the response variable, with line and plate nested within assay block fitted as random factors. Confidence intervals for the variance component estimates were calculated assuming that the sampling distribution of the variance can be approximated using a scaled chi-square distribution (Knight 2000). We used a general linear model to estimate the average rate of change of fitness per generation as a consequence of MA for each strain. We used fitness (μ_max_) as our response variable, and the average of the five replicate measures of each line (MA or pseudoline) as a single independent datapoint for each line when making comparisons among strains. Strain was fitted as a fixed factor, whereas the estimated number of generations of MA was fitted as a covariate. The ancestral pseudolines were included in the model as having zero generations of MA. We also fitted the interaction between strain and number of generations of MA to allow us to examine whether the change in fitness over time differs among strains. Because we expected the variance among lines to increase under MA, we included an additional parameter (varExp) to allow an exponential variance function structure in our model to account for such heterogeneity (Zuur et al. 2009). This parameter allows the variance to increase in relation to the value of the covariate in the model. Because our measure of fitness is a logarithmic measure, the rate of change of average fitness estimated from this model represents a change in relative fitness rather than absolute fitness.

To examine the relationship between fitness of MA lines estimated in liquid media and their growth rate on agar plates, we calculated the Pearson correlation coefficient between the average fitness measure for each MA line from our fitness assays and its average number of generations achieved growing on plates for four days. We calculated separate correlation coefficients for the MA lines derived from each ancestral strain. The individual *P*-values associated with these correlation coefficients were then combined using Fisher's method (Sokal and Rohlf 1994) to provide an overall hypothesis test of the relationship between fitness in the two conditions. The analysis was also carried out using the number of divisions achieved in five days growth as the measure of fitness on agar plates.

## Results

### NUMBER OF GENERATIONS OF MA

Based on pilot experiments in which the number of cells in growing colonies was counted under a microscope, we determined that wild-type colonies become visible after six generations of growth. Thus, a mutation that reduces the number of divisions below six per transfer period would be effectively lethal. Five of the ancestral strains (CC-1952, CC-2342, CC-2344, CC-2931, CC-2937) could achieve substantially more than six generations during the shortest period used in our transfer protocol (three days; Table2). For example, in the case of strain CC-2344, a reduction in fitness of about 21% would be required for division rate to drop below six generations per transfer. By the end of the experiment, the division rate of most MA lines had declined (Table S1), but still remained well above six generations per transfer. Furthermore, during the course of the experiment, we increased the minimum transfer period from three to four days, such that mutations that reduce the division rate by about 40% could still accumulate. However, in the case of strain CC-1373, the number of generations undergone by the ancestral strain was only ∼6.5 in a four-day period. The CC-1373 ancestral strain is therefore close to the threshold at which deleterious mutations with even small effects on growth rate would be effectively lethal under our transfer protocol, so the potential for deleterious mutations to accumulate in MA lines derived from this strain may have been limited. In addition, the rate of division of several of its MA lines had increased by the end of the experiment, suggesting that advantageous mutations may have accumulated (Table S1). Our MA protocol presumably resulted in more effective natural selection against slow growing lines in CC-1373 than lines derived from faster growing ancestral strains. We therefore exclude this strain from further analysis presented below. Estimates of mutational parameters including CC-1373 in the analysis are presented in Tables S2–S4.

**Table 2:** Estimates of numbers of generations undergone by each strain between transfers and over the entire MA experiment

Strain	Number of generations by ancestor in three days	Number of generations by ancestor in four days	Number of generations by ancestor in five days	Average number of generations by MA lines in four days (95% CIs)	Average number of generations by MA lines in five days (95% CIs)	Total number of generations of MA (95% CIs)
CC-1952	8.68	12.39	14.34	11.49 (11.00, 11.98)	13.12 (12.63, 13.60)	1021.12 (969.37, 1072.88)
CC-2342	10.90	12.72	13.46	11.43 (10.55, 12.31)	13.54 (12.76, 14.31)	1032.65 (942.31, 1122.98)
CC-2344	7.54	11.39	12.64	10.67 (10.11, 11.23)	12.22 (11.73, 12.71)	938.10 (912.50, 963.69)
CC-2931	9.50	12.06	13.48	11.08 (10.36, 11.79)	12.75 (11.87, 13.63)	1025.59 (985.90, 1065.28)
CC-2937	8.17	12.15	13.94	11.24 (10.77, 11.70)	13.08 (12.67, 13.48)	929.07 (854.31, 1003.83)

Estimates are shown for three-, four-, and five-day transfer periods for the ancestors. The three-day transfer period was only used in the early part of the study, and so three days measures were not made on the MA lines.

The number of generations per transfer also provides us with an estimate of the effective population size *N_e_*. This can be calculated as the harmonic mean of colony size throughout the transfer period. Over the longest transfer period used, the number of generations of growth was in the range 12–13, so *N_e_* ≈ 7. In a haploid organism, mutations for which *N_e_s* < 1 behave as effectively neutral, implying that selection would be effective against mutations with fitness effects of more than 15%.

We used the measures of growth on plates to estimate the total number of generations of MA undergone by each line. The average number of generations across all MA lines was 987 (Table2). The number of generations ranged from 1130 for one of the lines derived from CC-2342 to 404 generations for one of the CC-2937 MA lines that became contaminated during MA and had to be reestablished from frozen stock from an earlier time point (Table S1).

### CHANGES IN VARIANCE OF FITNESS AS A CONSEQUENCE OF MA

**Figure 1:**
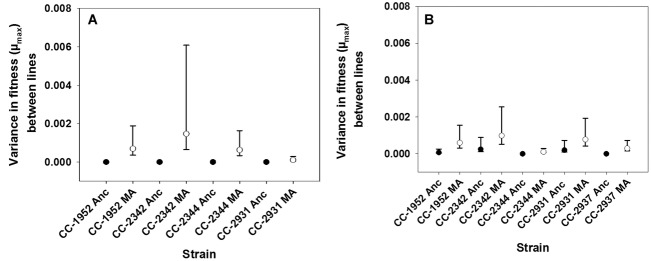
zAmong-line variance in fitness for each strain in the first (A) and second (B) fitness assays. Black circles are the among-line variance component for the ancestral pseudolines for each strain, and white circles are the among-line variance component for the MA lines for each strain. Bars are 95% confidence intervals.

For each ancestral strain, we estimated the variance component for fitness among the 15 MA lines and among the 9 ancestral pseudolines (Table3). In all cases, among-line variance components for the MA lines are significantly larger than the corresponding estimates for their ancestral strains (Fig.1, Table3). We calculated estimates of the new mutational variance per generation, *V*_m_, for each strain from the difference between the among-line variance component of the MA lines and that of the corresponding pseudolines divided by the estimated number of generations of MA (Table3). Estimates range from 1.03 × 10^−7^ to 1.42 × 10^−6^, with a mean of 5.42 × 10^−7^ across the two assays. Standardizing the square root of these variance estimates by the mean fitness of the ancestral strains yields estimates of the coefficient of mutational variation, *CV*_m ancestor_. These range from 0.19% for CC-2931 to 0.78% for CC-2342 in the first assay (Table3), with a mean across all strains and both fitness assays of 0.54%. Calculating the coefficient of mutational variation for each strain by scaling by the mean fitness of its MA lines *CV*_m MA_ produced similar values (Table3).

**Table 3:** Estimates of mutational variance for fitness assays

									Δ*V* per		*CV*_m ancestor_^5^ (% per		*CV*_m MA_^6^ (% per	
	Variance^1^ (95% CIs)				*F*-ratio test^2^		Δ*V*^3^		generation^4^ = *V*_m_		generation)		generation)	
Strain	First Anc	First MA	Second Anc	Second MA	First	Second	First	Second	First	Second	First	Second	First	Second
CC-1952	0 (0,0)	6.93×10^−4^ (3.56×10^−4^, 1.89×10^−3^)	6.99×10^−5^ (3.19×10^−5,^ 2.57×10^−4^)	5.99×10^−4^ (3.15×10^−4,^ 1.55×10^−3^)	NA^*^	8.57^*^	6.93×10^−4^	5.29×10^−4^	6.79×10^−7^	5.18×10^−7^	0.54	0.55	0.82	0.66
CC-2342	2.86×10^−17^ (1.30×10^−17,^ 1.05×10^−16^)	1.47×10^−3^ (6.42×10^−4^, 6.08×10^−3^)	2.43×10^−4^ (1.11×10^−4^, 8.90×10^−4^)	9.82×10^−4^ (5.16×10^−4,^ 2.55×10^−3^)	5.14×10^13^^*^	4.04^*^	1.47×10^−3^	7.39×10^−4^	1.42×10^−6^	7.16×10^−7^	0.78	0.65	1.06	0.85
CC-2344	0 (0,0)	6.3×10^−4^ (3.31×10^−4,^ 1.64×10^−3^)	0 (0,0)	1.09×10^−4^ (5.75×10^−5^, 2.84×10^−4^)	NA^*^	NA^*^	6.30×10^−4^	1.09×10^−4^	6.72×10^−7^	1.16×10^−7^	0.77	0.30	0.89	0.32
CC-2931	5.24×10^−17^ (2.39×10^−17^, 1.93×10^−16^)	1.061×10^−4^ (5.46×10^−5^, 2.89×10^−4^)	1.99×10^−4^ (9.07×10^−5^, 7.30×10^−4^)	7.76×10^−4^ (4.16×10^−4^, 1.93×10^−3^)	2.02×10^12^^*^	3.9^*^	1.06×10^−4^	5.77×10^−4^	1.03×10^−7^	5.63×10^−7^	0.19	0.58	0.25	0.71
CC-2937	−	−	7.15×10^−21^ (3.26×10^−21^, 2.62×10^−20^)	2.91×10^−4^ (1.56×10^−4^, 7.24×10^−4^)	−	4.07×10^16^^*^	−	2.91×10^−4^	−	3.13×10^−7^	−	0.51	−	0.54

Columns show estimates from the first and second replicate assays. Anc = ancestor; MA = mutation accumulation lines.

^1^Among MA line variance component.

^2^Ancestral among-pseudoline variance component divided by the between line MA variance component; the critical value = 3.006 with df = 15,9 (^*^significant difference at the 5% level between MA and ancestral variance).

^3^Change in the among-line variance component of fitness between the MA lines and their ancestors.

^4^Mutational heritability calculated as Δ*V* /number of generations.

^5^Coefficients of mutational variance calculated as the square root of Δ*V* per generation divided by the mean fitness of the ancestral lines.

^6^Coefficients of mutational variance calculated as the square root of Δ*V* per generation divided by the mean fitness of the MA lines.

### CHANGES IN MEAN FITNESS

**Figure 2:**
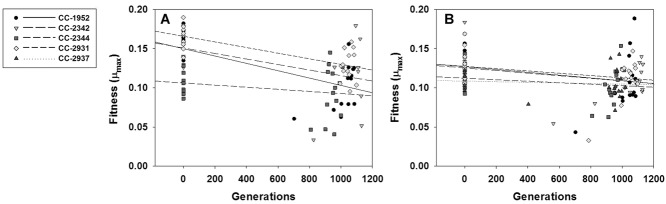
Estimates of fitness plotted against number of generations of mutation accumulation for all strains in the first (A) and second (B) fitness assays. The lines represent the linear regressions of fitness on the number of generations of mutation accumulation, with the ancestral pseudolines at 0 generations.

To examine the effect of MA on mean fitness, we modeled the average fitness of the MA lines as a function of the number of generations of MA. This analysis reveals that there is a negative relationship (*b*) between mean fitness and the number of generations of MA in both fitness assays (first assay: *b* = –3.5 × 10^−5^ μ_max_ generation^−1^, SE = 0.5 × 10^−5^, *F*_1,87_ = 57, *P* < 0.0001, Fig.2A; second assay: *b* = –1.3 × 10^−5^ μ_max_ generation^−1^, SE = 0.4 × 10^−5^, *F*_1,103_ = 8.42, *P* = 0.0014, Fig.2B). There is no evidence for variation in the effects of MA among strains, because the strain-by-number of generations interaction is nonsignificant in both the first (*F*_3,87_ = 2.18, *P* = 0.095) and second fitness assays (*F*_4,103_ = 0.45, *P* = 0.77). In the first fitness assay, strain CC-1952 showed the greatest decline in mean fitness (*b* = –5.1 × 10^−5^ μ_max_ generation^−1^, SE = 1.0 × 10^−5^), whereas CC-2344 had the smallest decline in mean fitness (*b* = –1.6 × 10^−5^ μ_max_ generation^−1^, SE = 1.1 × 10^−5^). In the second fitness assay, CC-1952 (*b* = –1.9 × 10^−5^ μ_max_ generation^−1^, SE = 0.9 × 10^−5^) was again estimated to have the greatest decline in mean fitness, whereas CC-2937 had the smallest decline (*b* = –0.3 × 10^−6^ μ_max_ generation^−1^, SE = 1.0 × 10^−5^).

### CORRELATION ACROSS ENVIRONMENTS

**Figure 3:**
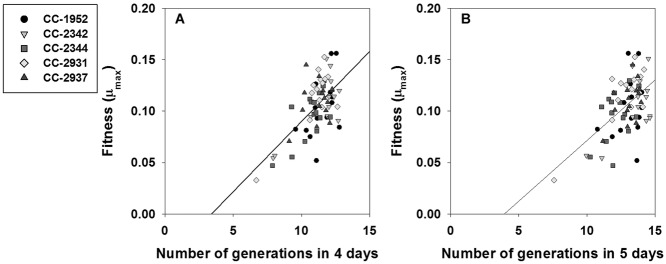
Correlation between the two measures of fitness: number of generations on agar plates and maximal specific growth rate (μ_max_) in liquid. (A) Number of generations in four days on agar plates versus μ_max_, (B) number of generations in five days on agar plates versus μ_max_.

Solid and liquid media represent very different growth environments for *C. reinhardtii*, even when they contain the same chemical components (Goho and Bell 2000). For each ancestral strain, we examined the correlation across MA lines between the estimates of growth rate in liquid media with estimates of the number of generations on solid agar media over four and five days. The correlation coefficients of all strains are positive (Pearson's *r* four-day: range = 0.258–0.806, mean = 0.606; five-day: range = 0.274–0.668, mean = 0.547), and the overall relationships were significant between average growth rate in liquid and four days of growth on agar plates (χ^2^ = 43.86, df = 10, *P* < 0.001, Fig.3A) and average growth rate in liquid and five days of growth on agar plates (χ^2^ = 35.49, df = 10, *P* < 0.001, Fig.3B).

### MUTATIONAL EFFECTS AND GENOMIC DELETERIOUS MUTATION RATE

We used the observed reductions in mean growth rate and increases in genetic variation to calculate BM estimates for the genomic rate of deleterious mutation (*U*) and the average effect of a deleterious mutation (E(*a*)). The BM method assumes that mutational effects are invariant, so estimates of *U* and the mean effect of a mutation are lower and upper bound estimates, respectively. SEs for these parameters were calculated using the Delta method (Lynch and Walsh 1997). Estimates of *U* ranged from 3.1 × 10^−5^ per generation for CC-2937 in the second fitness assay to 1.2 × 10^−2^ in CC-2931 in the first fitness assay (Table^4^), with an average estimate across both assays of 2.03 × 10^−3^. The average effect of a deleterious mutation (E(*a*)) varied from –2.91 × 10^−3^ μ_max_ generation^−1^ in strain CC-2931 in the first fitness assay to –1.0 × 10^−1^ in CC-2937 in the second fitness assay (Table4), with an average estimate across both assays of –4.07 × 10^−2^.

**Table 4:** Estimates of fitness and mutational parameters from the fitness assays

	Fitness (95% CI)				Δ*M* (× 10^−3^) per generation^1^ (95% CI [× 10^−3^ ])		*U* 2 (SE)		E(*a*)^3^ (SE)	
Strain	First Anc	First MA	Second Anc	Second MA	First	Second	First	Second	First	Second
CC-1952	0.153 (0.127, 0.178)	0.100 (0.079, 0.121)	0.130 (0.102, 0.158)	0.108 (0.071, 0.145)	−0.051 (−0.071, −0.030)	−0.019 (−0.037, −0.001)	3.80×10^−3^ (5.84×10^−3^)	6.84×10^−4^ (1.03×10^−3^)	−1.34×10^−2^ (1.30×10^−2^)	−2.75×10^−2^ (2.67×10^−2^)
CC-2342	0.153 (0.138, 0.168)	0.112 (0.061, 0.163)	0.130 (0.112, 0.148)	0.099 (0.078, 0.120)	−0.042 (−0.067, −0.017)	−0.017 (−0.035, +0.001)	1.24×10^−3^ (1.87×10^−3^)	3.92×10^−4^ (5.93×10^−4^)	−3.38×10^−2^ (3.28×10^−2^)	−4.27×10^−2^ (4.14×10^−2^)
CC-2344	0.107 (0.089, 0.125)	0.092 (0.062, 0.121)	0.114 (0.100, 0.128)	0.105 (0.086, 0.124)	−0.016 (−0.036, +0.005)	−0.011 (−0.031, +0.008)	3.68×10^−4^ (5.56×10^−4^)	1.13×10^−3^ (1.71×10^−3^)	−4.27×10^−2^ (4.12×10^−2^)	−1.02×10^−2^ (9.89×10^−3^)
CC-2931	0.166 (0.145, 0.187)	0.129 (0.110, 0.148)	0.130 (0.111, 0.149)	0.106 (0.088, 0.124)	−0.036 (−0.056, −0.015)	−0.017 (−0.034 +0.001)	1.22×10^−2^ (1.84×10^−2^)	4.89×10^−4^ (7.39×10^−4^)	−2.91×10^−3^ (2.82×10^−3^)	−3.39×10^−2^ (3.29×10 ^−2^)
CC-2937	−	−	0.109 (0.098, 0.121)	0.104 (0.090, 0.118)	−	−0.003 (−0.022, +0.016)	−	3.13×10^−5^ (4.73×10^−5^)	−	−1.00×10^−1^ (9.70×10^−2^)

Separate columns show estimates from the first and second replicate assays.

^1^Change in mean fitness per generation between the ancestral pseudolines (Anc) and MA lines (MA) for each strain.

^2^Bateman–Mukai estimates of the genomic deleterious mutation rate per generation.

^3^Bateman–Mukai estimates of the mean mutational effect per generation.

## Discussion

We estimated the rate of increase in genetic variance for fitness and rate of change of mean fitness in *C. reinhardtii* lines that had been maintained under relaxed selection for ∼1000 generations. In common with the majority of previous MA studies, an increase in variance among MA lines derived from a single ancestral strain was accompanied by a decline in mean fitness, consistent with lines accumulating predominantly deleterious mutations.

There is little evidence to suggest that the effects of MA varied among the different strains. The rate of decline of mean fitness did not significantly differ among MA lines derived from different ancestral strains. The standardized estimates of the increase in variance varied among strains within a single run of the fitness assay, but there was almost as much variation among the estimates for a single strain between two runs of the fitness assay as there was between strains. Averaging the BM estimates, the deleterious mutation rate (*U*) and average mutational effect (E(*a*)) across our five strains were 2.02 × 10^−3^ and 4.15 × 10^−2^, respectively.

The ancestral strains used in this study differed in initial fitness, and there are reasons to expect that this could affect the rate at which fitness declines as mutations accumulate. Strains with high starting fitness are already well adapted to the assay environment, and the potential number of beneficial mutations is likely to be small. In the case of less well adapted lines, the potential for mutations to improve fitness is greater, and this might lead to a higher ratio of beneficial to deleterious mutations, and a slower rate of fitness decline (Fisher 1930). Alternatively, there is empirical evidence that the mutation rate itself may depend on the fitness of an organism. For example, a study in *D. melanogaster* showed that individuals of reduced genetic quality have an elevated mutation rate (Sharp and Agrawal 2012). The lack of any differences among our strains does not support either of these predictions. However, although there was variation in fitness among all of our ancestral strains, it is likely that all of the strains were still reasonably well adapted to the laboratory environment, and so the range of initial fitness differences may have been too similar to allow us to detect such effects.

Selection of *C. reinhardtii* populations on either solid or liquid Bold's media leads to adaptation to the specific media (either solid or liquid), but minimal correlated adaptation to the other media (Goho and Bell 2000), implying that the genetic basis of adaptation to the two environments is very different. The fact that we see a positive correlation between the fitness of our MA lines when assayed in solid and liquid media suggests that many of the deleterious mutations that have accumulated have effects that are not environment specific. However, we note that both of these environments are relatively benign. It will be interesting to examine whether similar patterns are seen in more harsh or complex environments, where more genes are likely to be expressed.

Several of our MA lines could not be revived from their frozen stocks (13 in the first fitness assay and 4 in the second fitness assay, Table S1), so could not be included in the fitness assays. This may have affected our estimates of mutational parameters, as we cannot be certain whether the lines failed to grow because they had low fitness, or because they were killed during the freezing. Our estimates of the change in mean fitness per generation (Δ*M*) may therefore be underestimates if these lines had atypically low fitnesses. However, typically, one or two lines for each genotype failed to grow (Table S1), so we expect little systematic bias affecting estimates of Δ*M* or change in variance per generation (Δ*V*) between strains. Although several CC-2342 lines failed to grow in the first assay (seven, compared to two in the second assay), the estimates of Δ*M* were actually lower in the first assay than the second.

Our average estimates (across strains and fitness assays) of the standardized change in variance of fitness per generation (*CV*_m ancestor_) (0.54%) and mean fitness Δ*M* (–2.44 × 10^−5^) are lower than the average estimates for the multicellular eukaryotes *A. thaliana* (4.2%, –5 × 10^−3^), *D. melanogaster* (1.5%, –4.9 × 10^−3^), *C. elegans* (1.5%, –1.1 × 10^−3^), and *D. pulicaria* (no *CV*_m_ estimate, –1.4 × 10^−3^; Schaack et al. 2013), but higher than estimates for the unicellular organisms *S. cerevisiae* (0.1%, –5.5 × 10^−6^) and *E. coli* (0.02%, –2.5 × 10^−6^; see Tables5 and S5). The lower changes in the mean and variance of fitness per generation could be due to the fact that microbes undergo a single cell division per generation, whereas there are multiple germline cell divisions per generation in the multicellular species listed. It is also possible that differences are caused by the fact that mutations are accumulated in haploids and assayed in the hemizygous state in *C. reinhardtii* and *E. coli* and accumulated in diploids in *A. thaliana*, *D. melanogaster*, and *C. elegans* and usually assayed in the homozygous state. By controlling for the estimated number of germline divisions per generation, the ∼2180-fold variation in (ΔM across these MA studies is reduced to only ∼60-fold (see Table5), and the estimate for *C. reinhardtii* remains more similar to multicellular eukaryotes than unicellular microbes. Using published estimates of mutation rates and genome sizes, we can also scale values of (ΔM by the total number of mutations per cell division to get an estimate of (ΔM per genomic mutation. This allows comparison amongst species, independent of generation time, germline cell divisions per generation, genome size, or number of mutations. The variation in (ΔM per genomic mutation is only 7-sevenfold between species, suggesting that differences in (Δ*M* among MA studies in these organisms are driven largely by the cumulative number of mutations. If we control for the size of the protein coding fraction of each genome, the variation in Δ*M* among species is not further reduced. This is possibly because a sizeable fraction of each genome is functional noncoding DNA and the amount varies substantially between species (Lynch 2007). Similar calculations using *CV*_m_ show a similar pattern: much of the variation among species disappears if the size of the genome is taken into account and there is even less variation when controlling for the number of protein coding sites in the genome (Table S5). It should be noted that estimates of Δ*M* and *CV*_m_ are quite noisy (e.g., Δ*M* varies 36-fold within *D. melanogaster* alone) limiting the power of this analysis.

**Table 5:** Mutational and genomic properties of model organisms used for MA experiments

	μ (/bp^*^	Genome	Proteome size^1^	Coding		Cell div.	Mut./div.^*^	Mut./div.^*^				Δ*M*/genome^*^	Δ*M*/proteome^*^		
Organism	gen.)	size (bp)	(codons)	positions^2^	Ploidy	/gen.^3^	genome	proteome	*CV*_m_^4^	Δ*M*^4^	Δ*M*/div.	mut.	mut.	*U*	E(*a*)
*A. thaliana*	6.50×10^−9^	1.57×10^8^	1.34×10^7^	2.01×10^7^	2	35	0.0292	0.0037	4.15	−5.45×10^−3^	−1.56×10^−4^	−5.34×10^−3^	−4.17×10^−2^	0.027	−0.39
*C. elegans*	5.75×10^−9^	9.70×10^7^	1.00×10^7^	1.51×10^7^	2	9	0.062	0.0096	1.49	−1.07×10^−3^	−1.18×10^−4^	−1.91×10^−3^	−1.23×10^−2^	0.007	−0.25
*D. melanogaster*^5^	4.65×10^−9^	1.40×10^8^	7.10×10^6^	1.06×10^7^	2	36	0.018	0.0014	1.54	−4.87×10^−3^	−1.35×10^−4^	−7.15×10^−3^	−9.84×10^−2^	0.1	−0.14
*C. reinhardtii*	2.08×10^−10^	1.12×10^8^	1.31×10^7^	1.96×10^7^	1	1	0.0233	0.0041	0.54	−2.44×10^−5^	−2.44×10^−5^	−1.05×10^−3^	−5.97×10^−3^	0.002	−0.04
*S. cerevisiae*	3.30×10^−10^	1.25×10^7^	2.91×10^6^	4.36×10^6^	1	1	0.0041	0.0014	0.09	−5.45×10^−6^	−5.45×10^−6^	−1.32×10^−3^	−3.79×10^−3^	2.4×10^−5^	−0.13
*E. coli*	2.60×10^−10^	4.64×10^6^	1.32×10^6^	1.97×10^8^	1	1	0.0012	0.0005	0.02	−2.50×10^−6^	−2.50×10^−6^	−2.07×10^−3^	−4.88×10^−3^	1.7×10^−4^	−0.01

The organisms listed are those for which genomic and phenotypic outcomes of MA are available. The figures reported are the mean value for each species as summarized in Halligan and Keightley (2009 and references therein). Using available estimates of the mutation rate (μ), genome size, and proteome size, we calculate the change in fitness per mutation.

gen. = generations; div. = divisions.

^1^Proteome sizes were taken from Massey (2008) and references therein.

^2^The number of coding positions was estimated as two-thirds of CDS to account for synonymous changes.

^3^Cell divisions per generation were taken from Lynch (2010) and references therein.

^4^MA results were taken from Table1 of Halligan and Keightley (2009), which tabulated standardized parameter estimates from MA studies.

^5^Mean values for *D. melanogaster* exclude the extreme outlier values of Gong et al. (2005) (see Halligan and Keightley 2009 for details).

Our BM estimates of the average mutational effect, E(*a*), are generally lower than observed in other organisms, averaging –0.04 across strains and fitness assays, and ranging between –0.003 and –0.1. Even the largest estimate is lower than mean estimates from *A. thaliana* (–0.39), *C. elegans* (–0.25), *D. melanogaster* (–0.14), and *S. cerevisiae* (–0.13), but similar to *E. coli* (–0.012). This is consistent with new mutations in *C. reinhardtii* generally having small deleterious effects compared to multicellular organisms. However, if significant numbers of mutations were effectively lethal under our protocol, reducing fitness by more than 20%, or beneficial mutations became fixed, then the average mutational effect might be underestimated. We can compare mean BM estimates of E(*a*) for each species to our measured values of Δ*M*/mutation. E(*a*) is generally more negative, because it is estimated only from mutations that have detectable effects, which tend to be deleterious. Δ*M*/mutation, however, measures the effect for any mutation including neutral or beneficial mutations. The fact that it E(*a*) is more negative than Δ*M*/mutation across all studies (Table5) is consistent with a substantial fraction of mutations having small effects on fitness. We note that our average BM estimate of *U* (2.03 × 10^–3^) is higher than mean estimates for *S. cerevisiae* (2.4 × 10^−5^), *E. coli* (1.7 × 10^−4^), similar to *C. elegans* (7.0 × 10^−3^), but lower than *D. melanogaster* (1.0 × 10^−1^) and *A. thaliana* (2.7 × 10^−2^).

If we divide the average estimate of the genome-wide deleterious mutation rate *U* from the current study (2.03 × 10^−3^) by an estimate of the genome-wide mutation rate (3.62 × 10^−2^) that we previously obtained based on whole-genome sequencing (Ness et al. 2012), we infer that ∼5.6% of spontaneous mutations have detectable deleterious effect on fitness. A second direct estimate of the genome wide mutation rate, based on whole genome sequencing of a different strain of *C. reinhardtii* (7.57 × 10^−3^) is five times lower than our estimate (Sung et al. 2012), which suggests that ∼27% of all mutations are deleterious. Although these estimates are different, both imply that a substantial number of mutations have no measurable effect on fitness in the laboratory environment. This may be because many deleterious mutation have extremely small effects on fitness making them effectively undetectable by the approaches used in this article (Davies et al. 1999), but it could alternatively be explained if only a fraction of genes were expressed in *C. reinhardtii* in the fitness assay environment.

In summary, by measuring the response of *C. reinhardtii*, a microbe with a relatively large genome, to MA, we have provided evidence that the observed differences in mutational parameters between microbes and multicellular organisms are primarily a result of differences in genome size as it relates to the number of mutations expected to accumulate. We found no evidence that the initial level of adaptation of a strain affected the rate at which its fitness declined during MA when estimated in an environment to which all genotypes were reasonably well adapted. Whether this result extends to environments to which the strains were initially less adapted remains to be seen.

## DATA ARCHIVING

The doi for our data is 10.5061/dryad.s8330.

## Supporting Information

Disclaimer: Supplementary materials have been peer-reviewed but not copyedited.

**Table S2**Summary of the estimates of the number of divisions on plates over four and five days of growth and fitness in liquid media for each MA line and the ancestral strains. **Table S2**. Summary of estimates of numbers of generations undergone by each strain between transfers and over the entire MA experiment including CC-1373. **Table S3**. Summary of estimates of mutational variance for fitness assays including CC-1373. **Table S4**. Estimates of fitness and mutational parameters from the fitness assays including CC-1373. **Table S5**. Coefficient of mutation variance (*CV*m) and genomic properties of model organisms used for MA experiments.

## References

[b1] Adams MD, Celniker SE, Holt RA, Evans CA, Gocayne JD, Amanatides PG, Scherer SE, Li PW, Hoskins RA, Galle RF (2000). The genome sequence of *Drosophila melanogaster*. Science.

[b3] Baer CF, Shaw F, Steding C, Baurngartner M, Hawkins A, Houppert A, Mason N, Reed M, Simonelic K, Woodward W (2005). Comparative evolutionary genetics of spontaneous mutations affecting fitness in rhabditid nematodes. Proc. Natl. Acad. Sci. USA.

[b2] Baer CF, Miyamoto MM, Denver DR (2007). Mutation rate variation in multicellular eukaryotes: causes and consequences. Nat. Rev. Genet.

[b4] Baranyi J, Roberts TA (1994). A dynamic approach to predicting bacterial growth in food. Int. J. Food Microbiol.

[b5] Barrick JE, Kauth MR, Strelioff CC, Lenski RE (2010). rpoB mutants have increased evolvability in proportion to their fitness defects. Mol. Biol. Evol.

[b6] Bateman AJ (1959). The viability of near-normal irradiated chromosomes. Int. J. Rad. Biol.

[b8] Charlesworth B, Charlesworth D (1998). Some evolutionary consequences of deleterious mutations. Genetica.

[b7] Charlesworth B, Borthwick H, Bartolome C, Pignatelli P (2004). Estimates of the genomic mutation rate for detrimental alleles in *Drosophila melanogaster*. Genetics.

[b9] Crutchfield ALM, Diller KR, Brand JJ (1999). Cryopreservation of *Chlamydomonas reinhardtii* (Chlorophyta). Eur. J. Phycol.

[b10] Davies EK, Peters AD, Keightley PD (1999). High frequency of cryptic deleterious mutations in *Caenorhabditis elegans*. Science.

[b11] Dickinson WJ (2008). Synergistic fitness interactions and a high frequency of beneficial changes among mutations accumulated under relaxed selection in *Saccharomyces cerevisiae*. Genetics.

[b12] Drake JW, Charlesworth B, Charlesworth D, Crow JF (1998). Rates of spontaneous mutation. Genetics.

[b13] Fernandez J, López-Fanjul C (1996). Spontaneous mutational variances and covariances for fitness-related traits in *Drosophila melanogaster*. Genetics.

[b14] Fisher RA (1930). The genetical theory of natural selection.

[b15] Fry JD, Keightley PD, Heinsohn SL, Nuzhdin SV (1999). New estimates of the rates and effects of mildly deleterious mutation in *Drosophila melanogaster*. Proc. Natl. Acad. Sci. USA.

[b16] García-Dorado A, Monedero JL, López-Fanjul C (1998). The mutation rate and the distribution of mutational effects of viability and fitness in *Drosophila melanogaster*. Genetica.

[b17] Goffeau A, Barrell BG, Bussey H, Davis RW, Dujon B, Feldmann H, Galibert F, Hoheisel JD, Jacq C, Johnston M (1996). Life with 6000 genes. Science.

[b18] Goho S, Bell G (2000). The ecology and genetics of fitness in *Chlamydomonas*. IX. The rate of accumulation of variation of fitness under selection. Evolution.

[b66] Gong Y, Woodruff RC, Thompson JN (2005). Deleterious genomic mutation rate for viability in *Drosophila melanogaster* using concomitant sibling controls. Biol. Lett.

[b19] Haerty W, Ponting CP (2012). Mutations within lncRNAs are effectively selected against in fruitfly but not in human. Genome Biol.

[b20] Halligan DL, Keightley PD (2009). Spontaneous mutation accumulation studies in evolutionary genetics. Annu. Rev. Ecol. Evol. Syst.

[b21] Halligan DL, Kousathanas A, Ness RW, Harr B, Eory L, Keane TM, Adams DJ, Keightley PD (2013). Contributions of protein-coding and regulatory change to adaptive molecular evolution in murid rodents. PLoS Genet.

[b22] Hartfield M, Keightley PD (2012). Current hypotheses for the evolution of sex and recombination. Integ. Zoo.

[b23] Joseph SB, Hall DW (2004). Spontaneous mutations in diploid *Saccharomyces cerevisiae*: more beneficial than expected. Genetics.

[b25] Keightley PD, Caballero A (1997). Genomic mutation rates for lifetime reproductive output and lifespan in *Caenorhabditis elegans*. Proc. Natl. Acad. Sci. USA.

[b26] Keightley PD, Eyre-Walker A (1999). Terumi Mukai and the riddle of deleterious mutation rates. Genetics.

[b27] Keightley PD, Eyre-Walker A (2010). What can we learn about the distribution of fitness effects of new mutations from DNA sequence data. Philos. Trans. R. Soc. B.

[b28] Keightley PD, Lynch M (2003). Toward a realistic model of mutations affecting fitness. Evolution.

[b30] Keightley PD, Trivedi U, Thomson M, Oliver F, Kumar S, Blaxter M (2009). Analysis of the genome sequences of three *Drosophila melanogaster* spontaneous mutation accumulation lines. Genome Res.

[b29] Keightley PD, Ness RW, Halligan DL, Haddrill PR (2014). Estimation of the spontaneous mutation rate per nucleotide site in a *Drosophila melanogaster* full-sib family. Genetics.

[b31] Kibota TT, Lynch M (1996). Estimate of the genomic mutation rate deleterious to overall fitness in *E. coli*. Nature.

[b32] Knight K (2000). Mathematical statistics.

[b33] Kondrashov AS (1988). Deleterious mutations and the evolution of sexual reproduction. Nature.

[b34] Koonin EV (2003). Comparative genomics, minimal gene-sets and the last universal common ancestor. Nat. Rev. Microbiol.

[b35] Liss M, Kirk DL, Beyser K, Fabry S (1997). Intron sequences provide a tool for high-resolution phylogenetic analysis of volvocine algae. Curr. Genet.

[b36] Loewe L, Hill WG (2010). The population genetics of mutations: good, bad and indifferent. Philos. Trans. R. Soc. B.

[b37] Lukjancenko O, Wassenaar TM, Ussery DW (2010). Comparison of 61 sequenced *Escherichia coli* genomes. Microb. Ecol.

[b38] Lynch M (2007). The origins of genome architecture.

[b39] Lynch M (2010). Evolution of the mutation rate. Trends Genet.

[b41] Lynch M, Walsh B (1997). Genetics and the analysis of quantitative traits.

[b40] Lynch M, Blanchard J, Houle D, Kibota T, Schultz S, Vassilieva L, Willis J (1999). Perspective: spontaneous deleterious mutation. Evolution.

[b42] Massey SE (2008). The proteomic constraint and its role in molecular evolution. Mol. Biol. Evol.

[b43] Matic I, Radman M, Taddei F, Picard B, Doit C, Bingen E, Denamur E, Elion J (1997). Highly variable mutation rates in commensal and pathogenic *Escherichia coli*. Science.

[b44] Merchant SS, Prochnik SE, Vallon O, Harris EH, Karpowicz SJ, Witman GB, Terry A, Salamov A, Fritz-Laylin LK, Maréchal-Drouard L (2007). The *Chlamydomonas* genome reveals the evolution of key animal and plant functions. Science.

[b45] Mukai T (1964). Genetic structure of natural populations of *Drosophila melanogaster*. 1. Spontaneous mutation rate of polygenes controlling viability. Genetics.

[b46] Mukai T, Chigusa SI, Crow JF, Mettler LE (1972). Mutation rate and dominance of genes affecting viability in *Drosophila melanogaster*. Genetics.

[b47] Ness RW, Morgan AD, Colegrave N, Keightley PD (2012). Estimate of the spontaneous mutation rate in *Chlamydomonas reinhardtii*. Genetics.

[b48] Otto SP (2009). The evolutionary enigma of sex. Am. Nat.

[b49] R Core Team (2013). R: a language and environment for statistical computing.

[b50] Schaack S, Allen DE, Latta LC, Morgan KK, Lynch M (2013). The effect of spontaneous mutations on competitive ability. J. Evol. Biol.

[b51] Schoen DJ (2005). Deleterious mutation in related species of the plant genus *Amsinckia* with contrasting mating systems. Evolution.

[b52] Schrider DR, Houle D, Lynch M, Hahn MW (2013). Rates and genomic consequences of spontaneous mutational events in *Drosophila melanogaster*. Genetics.

[b53] Schultz ST, Lynch M, Willis JH (1999). Spontaneous deleterious mutation in *Arabidopsis thaliana*. Proc. Natl. Acad. Sci. USA.

[b54] Sharp NP, Agrawal AF (2012). Evidence for elevated mutation rates in low quality genotypes. Proc. Natl. Acad. Sci. USA.

[b55] Shaw RG, Byers DL, Darmo E (2000). Spontaneous mutational effects on reproductive traits of *Arabidopsis thaliana*. Genetics.

[b56] Smith DR, Lee RW (2008). Nucleotide diversity in the mitochondrial and nuclear compartments of *Chlamydomonas reinhardtii*: investigating the origins of genome architecture. BMC Evol. Biol.

[b57] Sokal RR, Rohlf FJ (1994). Biometry.

[b58] Sousa A, Magalhães S, Gordo I (2012). Cost of antibiotic resistance and the geometry of adaptation. Mol. Biol. Evol.

[b59] Sundin GW, Weigand MR (2007). The microbiology of mutability. FEMS Microbiol. Lett.

[b60] Sung W, Ackerman MS, Miller SF, Doak TG, Lynch M (2012). Drift-barrier hypothesis and mutation rate evolution. Proc. Natl. Acad. Sci. USA.

[b24] The Arabadopsis GenomeInitiative (2000). Analysis of the genome sequence of the flowering plant *Arabidopsis thaliana*. Nature.

[b61] The *C. elegans* (1998). Genome sequence of the nematode *C. elegans*: a platform for investigating biology. Science.

[b62] Torgerson DG, Boyko AR, Hernandez RD, Indap A, Hu X, White TJ, Sninsky JJ, Cargill M, Adams MD, Bustamante CD (2009). Evolutionary processes acting on candidate cis-regulatory regions in humans inferred from patterns of polymorphism and divergence. PLoS Genet.

[b63] Winzeler EA, Shoemaker DD, Astromoff A, Liang H, Anderson K, Andre B, Bangham R, Benito R, Boeke JD, Bussey H (1999). Functional characterization of the *S. cerevisiae* genome by gene deletion and parallel analysis. Science.

[b64] Zeyl C, DeVisser J (2001). Estimates of the rate and distribution of fitness effects of spontaneous mutation in *Saccharomyces cerevisiae*. Genetics.

[b65] Zuur AF, Ieno EN, Walker NJ, Saveliev AA, Smith GM (2009). Mixed effects models and extensions in ecology with R.

