# Supplementary material for: SPONTANEOUS MUTATION ACCUMULATION IN MULTIPLE STRAINS OF THE GREEN ALGA, CHLAMYDOMONAS REINHARDTII
Source: Evolution. 2014 Jul 9;68(9):2589–602. doi: 10.1111/evo.12448 (PMC4277324; doi:10.1111/evo.12448)
Supplement: Supplementary file 1 — Table S2Summary of the estimates of the number of divisions on plates over four and five days of growth and fitness in liquid media for each MA line and the ancestral strains. Table S2. Summary of estimates of numbers of generations undergone by each strain between transfers and over the entire MA experiment including CC-1373. Table S3. Summary of estimates of mutational variance for fitness assays including CC-1373. Table S4. Estimates of fitness and mutational parameters from the fitness assays including CC-1373. Table S5. Coefficient of mutation variance (CVm) and genomic properties of model organisms used for MA experiments. [file evo0068-2589-sd1.pdf]

Supporting Information

Table S1

Summary of the estimates of the number of divisions on plates over 4 and 5 days of growth and fitness in liquid media for each MA line and the ancestral strains. The μ_{max} shown for the ancestors is the average of 9 pseudolines. Missing values correspond to cases where the lines failed to grow from their frozen stocks. In cases where a replicate assay failed, the standard error for G cannot be calculated, and is indicated by (*) in the table.

Line	Total transfers of MA	Number of generations on plates in 4 days (se)	Number of generations on plates in 5 days (se)	Total estimated generations of MA	μ_{max} 1st fitness assay	μ_{max} 2nd fitness assay	Average μ_{max} of both fitness assays
**1373 Anc**	0	6.47 (*)	10.20 (0.77)	0	0.082	0.070	0.076
**CC-1373 1**	85	8.08 (*)	10.29(*)	771	0.095	0.082	0.088
**CC-1373 2**	74	8.06 (*)	10.87 (0.35)	683	0.060	0.073	0.066
**CC-1373 3**	72	8.59 (*)	11.13 (1.69)	678	0.104	0.094	0.099
**CC-1373 4**	80	6.79 (*)	9.59 (*)	689	0.045	0.079	0.062
**CC-1373 5**	77	6.97 (*)	10.68 (1.71)	693	0.080	0.110	0.095
**CC-1373 6**	84	8.36 (0.40)	9.66 (0.99)	749	0.082	0.081	0.081
**CC-1373 7**	82	8.90 (0.57)	10.77 (0.40)	766	0.093	0.088	0.090
**CC-1373 8**	80	8.55 (0.27)	10.02 (1.19)	725	0.086	0.080	0.083
**CC-1373 9**	79	-	-	-	-	-	-
**CC-1373 10**	82	5.86 (*)	7.29 (*)	630	0.057	0.061	0.059
**CC-1373 11**	82	8.79 (0.75)	10.24 (1.18)	751	0.108	0.058	0.083
**CC-1373 12**	79	-	-	-	-	-	-
**CC-1373 13**	82	9.39 (0.86)	10.78 (0.45)	773	0.066	0.087	0.077
**CC-1373 14**	81	6.41 (*)	11.03 (1.11)	732	0.057	0.067	0.062
**CC-1373 15**	23	8.55(0.58)	11.22 (0.23)	198	0.093	0.058	0.076

Line	Total transfers of MA	Number of generations on plates in 4 days (se)	Number of generations on plates in 5 days (se)	Total estimated generations of MA	μ_{max} 1st fitness assay	μ_{max} 2nd fitness assay	Average μ_{max} of both fitness assays
**1952 Anc**	0	12.39 (1.92)	14.34 (0.63)	0	0.153	0.130	0.141
**CC-1952 1**	85	11.86 (1.40)	13.83 (0.73)	1074	-	0.094	0.094
**CC-1952 2**	85	12.11 (1.32)	14.03 (0.57)	1083	0.125	0.111	0.118
**CC-1952 3**	85	12.18 (1.43)	13.79 (0.24)	1077	0.124	0.188	0.156
**CC-1952 4**	85	9.57 (0.44)	10.77 (0.31)	954	0.072	0.093	0.082
**CC-1952 5**	58	11.09 (*)	13.66 (*)	703	0.060	0.043	0.052
**CC-1952 6**	85	12.25 (1.35)	13.28 (0.13)	1064	0.112	0.116	0.114
**CC-1952 7**	85	12.24 (1.42)	12.71 1.02)	1048	0.126	0.091	0.108
**CC-1952 8**	85	10.64 (*)	11.86 (*)	1000	0.063	0.088	0.075
**CC-1952 9**	85	-	-	-	-	-	-
**CC-1952 10**	85	11.15 (0.55)	13.26 (0.49)	1047	0.079	0.107	0.093
**CC-1952 11**	85	11.01 (0.37)	14.02 (0.53)	1066	0.115	0.091	0.103
**CC-1952 12**	85	11.06 (0.75)	13.19 (0.28)	1044	0.112	0.141	0.127
**CC-1952 13**	84	10.35 (1.19)	12.47 (0.17)	1002	0.079	0.083	0.081
**CC-1952 14**	85	12.78 (1.25)	13.73 (0.05)	1085	0.079	0.089	0.084
**CC-1952 15**	84	12.55 1.59)	13.03 (0.51)	1049	0.156	0.157	0.156

Line	Total transfers of MA	Number of generations on plates in 4 days (se)	Number of generations on plates in 5 days (se)	Total estimated generations of MA	μ_{max} 1st fitness assay	μ_{max} 2nd fitness assay	Average μ_{max} of both fitness assays
**2342 Anc**	0	12.72 (0.06)	13.46 (0.81)	0	0.153	0.130	0.142
**CC-2342 1**	85	12.39 (0.01)	13.75 (0.34)	1109	0.121	0.108	0.114
**CC-2342 2**	85	-	-	-	-	-	-
**CC-2342 3**	83	10.68 (0.30)	13.36 (0.59)	1048	-	0.095	0.095
**CC-2342 4**	85	12.12 (0.09)	13.34 (0.35)	1093	0.179	0.109	0.144
**CC-2342 5**	84	12.77 (0.69)	14.42 (0.14)	1121	-	0.120	0.120
**CC-2342 6**	85	-	-	-	-	-	-
**CC-2342 7**	85	11.90 (0.26)	14.63 (0.55)	1126	-	0.095	0.095
**CC-2342 8**	50	7.92 (0.47)	11.10 (0.74)	562	-	0.054	0.054
**CC-2342 9**	73	12.02 (1.12)	14.35 (0.33)	959	0.126	0.102	0.114
**CC-2342 10**	85	12.70 (0.47)	14.35 (0.13)	1130	0.052	0.130	0.091
**CC-2342 11**	85	12.16 (0.52)	13.88 (0.78)	1109	0.126	0.132	0.129
**CC-2342 12**	75	8.04 (*)	9.96 (0.50)	826	0.034	0.080	0.057
**CC-2342 13**	85	12.05 (0.01)	14.62 (1.03)	1128	0.090	0.098	0.094
**CC-2342 14**	84	12.02 (0.29)	13.76 (0.27)	1091	-	0.104	0.104
**CC-2342 15**	85	11.83 (0.43)	14.50 (1.37)	1122	0.162	0.140	0.151

Line	Total transfers of MA	Number of generations on plates in 4 days (se)	Number of generations on plates in 5 days (se)	Total estimated generations of MA	μ_{max} 1st fitness assay	μ_{max} 2nd fitness assay	Average μ_{max} of both fitness assays
**2344 Anc**	0	11.39 (0.15)	12.64 (0.09)	0	0.107	0.114	0.110
**CC-2344 1**	84	10.26 (0.28)	11.40 (0.31)	912	0.079	0.063	0.071
**CC-2344 2**	84	10.61 (0.31)	11.61 (0.21)	923	0.130	0.093	0.112
**CC-2344 3**	84	12.12 (0.35)	13.62 (0.49)	1000	0.065	0.124	0.094
**CC-2344 4**	79	9.30 (0.32)	10.23 (0.32)	809	0.047	0.064	0.055
**CC-2344 5**	83	11.12 (0.13)	13.04 (0.74)	958	0.040	0.121	0.081
**CC-2344 6**	85	10.91 (0.36)	12.77 (0.03)	971	0.083	0.110	0.097
**CC-2344 7**	85	11.65 (0.04)	13.10 (0.28)	991	0.106	0.154	0.130
**CC-2344 8**	85	11.59 (0.35)	13.81 (0.12)	1010	0.127	0.122	0.125
**CC-2344 9**	85	7.88 (0.38)	11.91 (2.31)	901	0.047	-	0.047
**CC-2344 10**	83	10.72 (0.73)	11.79 (*)	917	0.120	0.098	0.109
**CC-2344 11**	84	9.27 (0.29)	11.08 (0.55)	890	-	0.104	0.104
**CC-2344 12**	83	11.36 (0.32)	12.65 (0.38)	950	0.094	0.094	0.094
**CC-2344 13**	85	10.93 (0.04)	12.43 (0.53)	961	0.100	0.117	0.108
**CC-2344 14**	83	11.31 (0.28)	12.01 (0.30)	931	0.145	0.090	0.118
**CC-2344 15**	85	11.07 (0.36)	11.83 (0.26)	947	0.118	0.078	0.098

Line	Total transfers of MA	Number of generations on plates in 4 days (se)	Number of generations on plates in 5 days (se)	Total estimated generations of MA	μ_{max} 1st fitness assay	μ_{max} 2nd fitness assay	Average μ_{max} of both fitness assays
**2931 Anc**	0	12.06 (0.27)	13.48 (*)	0	0.166	0.130	0.148
**CC-2931 1**	84	11.42 (0.15)	13.44 (0.23)	1050	0.141	0.120	0.131
**CC-2931 2**	85	11.49 (0.07)	13.24 (0.45)	1057	0.096	0.113	0.104
**CC-2931 3**	82	10.89 (0.13)	12.88 (0.21)	1000	0.128	0.123	0.126
**CC-2931 4**	83	11.54 (0.01)	12.48 (0.07)	1011	0.129	0.125	0.127
**CC-2931 5**	84	10.60 (1.15)	11.84 (1.18)	994	0.105	0.078	0.091
**CC-2931 6**	85	11.94 (0.64)	13.94 (0.20)	1083	0.142	0.125	0.134
**CC-2931 7**	85	11.69 (1.33)	13.51 (0.26)	1067	0.159	0.147	0.153
**CC-2931 8**	86	-	-	-	-	0.023	0.023
**CC-2931 9**	85	11.24 (0.22)	14.18 (0.79)	1079	0.152	0.129	0.141
**CC-2931 10**	85	12.64 (0.65)	14.09 (1.24)	1098	0.104	0.105	0.104
**CC-2931 11**	85	11.72 (0.11)	11.84 (0.43)	1021	0.151	0.111	0.131
**CC-2931 12**	81	6.70 (*)	7.59 (*)	787	-	0.033	0.033
**CC-2931 13**	84	11.13 (0.11)	13.28 (0.07)	1041	0.121	0.122	0.121
**CC-2931 14**	84	11.32 (0.13)	12.28 (0.67)	1016	0.122	0.100	0.111
**CC-2931 15**	84	10.76 (1.45)	13.86 (0.15)	1053	0.117	0.118	0.118

Line	Total transfers of MA	Number of generations on plates in 4 days (se)	Number of generations on plates in 5 days (se)	Total estimated generations of MA	μ_{max} 1st fitness assay	μ_{max} 2nd fitness assay	Average μ_{max} of both fitness assays
**2937 Anc**	0	12.14 (0.27)	13.94 (0.34)	0	0.065	0.110	0.088
**CC-2937 1**	75	11.29 (0.83)	13.73 (0.38)	925	0.130	0.138	0.134
**CC-2937 2**	81	11.68 (0.19)	13.06 (0.36)	991	0.114	0.112	0.113
**CC-2937 3**	81	12.23 (0.78)	12.96 (0.39)	996	0.145	0.096	0.121
**CC-2937 4**	81	11.92 (0.90)	13.97 (0.56)	1019	0.117	0.101	0.109
**CC-2937 5**	78	11.79 (0.37)	12.96 (0.17)	951	0.108	0.094	0.101
**CC-2937 6**	79	11.58 (0.60)	13.62 (1.10)	977	0.144	0.096	0.120
**CC-2937 7**	79	11.13 (0.58)	12.98 (0.03)	954	0.098	0.071	0.085
**CC-2937 8**	39	9.10 (0.52)	11.17 (0.09)	404	0.063	0.079	0.071
**CC-2937 9**	77	12.25 (0.42)	13.85 (0.35)	967	0.122	0.121	0.122
**CC-2937 10**	78	11.33 (0.82)	13.10 (0.04)	949	0.111	0.099	0.105
**CC-2937 11**	77	11.52 (0.37)	13.80 (0.40)	956	0.133	0.102	0.117
**CC-2937 12**	81	10.34 (0.57)	13.36 (*)	981	0.147	0.143	0.145
**CC-2937 13**	83	10.22 (0.64)	11.97 (0.44)	965	0.113	0.123	0.118
**CC-2937 14**	79	12.07 (0.77)	13.61 (0.84)	984	-	0.088	0.088
**CC-2937 15**	79	10.08 (0.68)	12.05 (*)	916	0.102	0.100	0.101

Table S2

Summary of estimates of numbers of generations undergone by each strain between transfers and over the entire MA experiment including CC-1373. Estimates are shown for three, four and five day transfer periods for the ancestors. The three day transfer period was only used in the early part of the study, and so three days measures were not made on the MA lines.

Strain	Number of generations of ancestor in 3 days	Number of generations of ancestor in 4 days	Number of generations of ancestor in 5 days	Average number of generations of MA lines in 4 days (95% CIs)	Average number of generations of MA lines in 5 days (95% CIs)	Total number of generations of MA (95% CIs)
**CC-1373**	7.11	6.47	10.20	7.95 (7.36, 8.53)	10.28 (9.71, 10.84)	679.89 (597.75, 762.02)
**CC-1952**	8.68	12.39	14.34	11.49 (11.00, 11.98)	13.12 (12.63, 13.60)	1021.12 (969.37, 1072.88)
**CC-2342**	10.90	12.72	13.46	11.43 (10.55, 12.31)	13.54 (12.76, 14.31)	1032.65 (942.31, 1122.98)
**CC-2344**	7.54	11.39	12.64	10.67 (10.11, 11.23)	12.22 (11.73, 12.71)	938.10 (912.50, 963.69)
**CC-2931**	9.50	12.06	13.48	11.08 (10.36, 11.79)	12.75 (11.87, 13.63)	1025.59 (985.90, 1065.28)
**CC-2937**	8.17	12.15	13.94	11.24 (10.77, 11.70)	13.08 (12.67, 13.48)	929.07 (854.31, 1003.83)

Table S3

Summary of estimates of mutational variance for fitness assays including CC-1373. Separate columns show estimates from the 1st and 2nd replicate assays (Anc = ancestor, MA= mutation accumulation lines).

Table S3

Strain	Variance (95% CIs)				F-ratio test		ΔV		ΔV per generation = V_m		CV_m Ancestor (% per generation)		CV_m MA (% per generation)	
	1st Anc	1st MA	2nd Anc	2nd MA	1st	2nd	1st	2nd	1st	2nd	1st	2nd	1st	2nd
**CC-1373**	0 (0, 0)	3.34×10^{-4} (1.72×10^{-4}, 9.1×10^{-4})	5.28×10^{-5} (2.41×10^{-5}, 1.94×10^{-4})	1.22×10^{-4} (6.28×10^{-5}, 3.33×10^{-4})	NA*	2.31	3.34×10^{-4}	6.92×10^{-5}	4.91×10^{-7}	1.02×10^{-7}	0.86	0.46	0.89	0.41
**CC-1952**	0 (0,0)	6.93×10^{-4} (3.56×10^{-4}, 1.89×10^{-3})	6.99×10^{-5} (3.19×10^{-5}, 2.57×10^{-4})	5.99×10^{-4} (3.15×10^{-4}, 1.55×10^{-3})	NA*	8.57*	6.93×10^{-4}	5.29×10^{-4}	6.79×10^{-7}	5.18×10^{-7}	0.54	0.55	0.82	0.66
**CC-2342**	2.86×10^{-17} (1.30×10^{-17}, 1.05×10^{-16})	1.47×10^{-3} (6.42×10^{-4}, 6.08×10^{-3})	2.43×10^{-4} (1.11×10^{-4}, 8.90×10^{-4})	9.82×10^{-4} (5.16×10^{-4}, 2.55×10^{-3})	5.14×10^{13}*	4.04*	1.47×10^{-3}	7.39×10^{-4}	1.42×10^{-6}	7.16×10^{-7}	0.78	0.65	1.06	0.85
**CC-2344**	0 (0,0)	6.3×10^{-4} (3.31×10^{-4}, 1.64×10^{-3})	0 (0,0)	1.09×10^{-4} (5.75×10^{-5}, 2.84×10^{-4})	NA*	NA*	6.30×10^{-4}	1.09×10^{-4}	6.72×10^{-7}	1.16×10^{-7}	0.77	0.30	0.89	0.32
**CC-2931**	5.24×10^{-17} (2.39×10^{-17}, 1.93×10^{-16})	1.06×10^{-4} (5.46×10^{-5}, 2.89×10^{-4})	1.99×10^{-4} (9.07×10^{-5}, 7.30×10^{-4})	7.76×10^{-4} (4.16×10^{-4}, 1.93×10^{-3})	2.02×10^{12}*	3.9*	1.06×10^{-4}	5.77×10^{-4}	1.03×10^{-7}	5.63×10^{-7}	0.19	0.58	0.25	0.71
**CC-2937**	-	-	7.15×10^{-21} (3.26×10^{-21}, 2.62×10^{-20})	2.91×10^{-4} (1.56×10^{-4}, 7.24×10^{-4})	-	4.07×10^{16}*	-	2.91×10^{-4}	-	3.13×10^{-7}	-	0.51	-	0.54

[1] among MA line variance component.
[2] ancestral among pseudoline variance component divided by the between line MA variance component; the critical value = 3.006 with df 15,9 (* = significant difference at the 5% level between MA and ancestral variance).
[3] change in the among line variance component of fitness between the MA lines and their ancestors.
[4] Mutational heritability calculated as ΔV /number of generations.
[5] coefficients of mutational variance calculated as the square root of ΔV per generation divided by the mean fitness of the ancestral lines
[6] coefficients of mutational variance calculated as the square root of ΔV per generation divided by the mean fitness of the MA lines

Table S4

Estimates of fitness and mutational parameters from the fitness assays including CC-1373. Separate columns show estimates from the 1st and 2nd replicate assays.

Strain	Fitness (95% CIs)				ΔM (x 10^{-3}) per generation[1] (95% CIs (x 10^{-3}))		U[2] (se)		$E(a)$[3] (se)	
	1st Anc	1st MA	2nd Anc	2nd MA	1st	2nd	1st	2nd	1st	2nd
**CC-1373**	0.082 (0.068, 0.096)	0.079 (0.068, 0.090)	0.069 (0.058, 0.081)	0.078 (0.066, 0.090)	-0.007 (-0.031, +0.017)	+0.018 (-0.006, +0.042)	1.01×10^{-4} (1.53×10^{-4})	*	-6.98×10^{-2} (6.77×10^{-2})	$+5.71 \times 10^{-3}$ (5.54×10^{-3})
**CC-1952**	0.153 (0.127, 0.178)	0.100 (0.079, 0.121)	0.130 (0.102, 0.158)	0.108 (0.071, 0.145)	-0.051 (-0.071, -0.031)	-0.019 (-0.036, -0.002)	3.86×10^{-3} (4.55×10^{-3})	7.08×10^{-4} (1.07×10^{-3})	-1.33×10^{-2} (1.29×10^{-2})	-2.70×10^{-2} (2.62×10^{-2})
**CC-2342**	0.153 (0.138, 0.168)	0.112 (0.061, 0.163)	0.130 (0.112, 0.148)	0.099 (0.078, 0.120)	-0.042 (-0.066, -0.018)	-0.017 (-0.034, 0.001)	1.26×10^{-3} (1.91×10^{-3})	4.27×10^{-4} (6.46×10^{-4})	-3.36×10^{-2} (3.26×10^{-2})	-4.09×10^{-2} (3.97×10^{-2})
**CC-2344**	0.107 (0.089, 0.125)	0.092 (0.062, 0.121)	0.114 (0.100, 0.128)	0.105 (0.086, 0.124)	-0.016 (-0.036, +0.004)	-0.011 (-0.030, +0.007)	3.71×10^{-4} (5.61×10^{-4})	1.13×10^{-3} (1.71×10^{-3})	-4.26×10^{-2} (4.13×10^{-2})	-1.01×10^{-2} (9.80×10^{-2})
**CC-2931**	0.166 (0.145, 0.187)	0.129 (0.110, 0.148)	0.130 (0.111, 0.149)	0.106 (0.088, 0.124)	-0.036 (-0.055, -0.016)	-0.017 (-0.034, 0.000)	1.22×10^{-2} (1.84×10^{-2})	5.02×10^{-4} (7.59×10^{-4})	-2.91×10^{-3} (2.72×10^{-3})	-3.35×10^{-2} (3.25×10^{-2})
**CC-2937**	-	-	0.109 (0.098, 0.121)	0.104 (0.090, 0.118)	-	-0.003 (-0.021, +0.014)	-	3.13×10^{-5} (4.73×10^{-5})	-	-9.16×10^{-2} (8.89×10^{-2})

[1] change in mean fitness per generation between the ancestral pseudolines (Anc) and the MA lines (MA) for each strain.
[2] Bateman-Mukai estimates of the genomic deleterious mutation rate per generation, se calculated using the delta method
[3] Bateman-Mukai estimates of the mean mutational effect per generation, se calculated using the delta method.

Table S5. Coefficient of mutation variance (CV_m) and genomic properties of model organisms used for MA experiments. The organisms listed are those for which genomic and phenotypic outcomes of MA are available. The figures reported for CV_m are the mean value for each species as summarised in Halligan and Keightley (2009 and references therein). Using available estimates of the mutation rate, genome size and, proteome size we calculate the variance in fitness contributed per mutation.

Organism	μ (/bp*gen.)	genome size (bp)	Coding positions [1]	Ploidy	cell div. / gen.[2]	Mut. / div. * genome	Mut./div.* proteome	CV_m	CV_m / div.	CV_m / genome *mut.	CV_m / proteome *mut.
Arabidopsis thaliana	6.50 x 10^{-9}	157,000,000	20,098,557.72	2	35	0.0292	0.0037	4.15	0.119	4.1	31.8
Caenorhabditis elegans	5.75 x 10^{-9}	97,000,000	15,058,140.93	2	9	0.062	0.0096	1.49	0.165	2.7	17.2
Drosophila melanogaster [3]	4.65 X 10^{-9}	139,500,000	10,649,322.34	2	36	0.018	0.0014	1.54	0.043	2.4	31.0
Chlamydomonas reinhardtii	2.08 x 10^{-10}	112,000,000	19,641,831.08	1	1	0.0233	0.0041	0.54	0.540	23.2	132.2
Saccharomyces cerevisiae	3.30 x 10^{-10}	12,495,682	4,357,469.27	1	1	0.0041	0.0014	0.09	0.087	21.1	60.5
E. coli	2.60 x 10^{-10}	4,639,675	1,972,335.83	1	1	0.0012	0.0005	0.02	0.020	16.6	39.0

[1] Proteome sizes were taken from Massey 2008 and references therein. The number of coding positions was estimated as 2/3 of CDS to account for synonymous changes

[2] Cell divisions per generation were taken from Lynch (2010) and references therein.

References

Lynch, M. 2010. Evolution of the mutation rate. Trends Genet. 26:345-352.

Halligan, D. L. and P. D. Keightley. 2009. Spontaneous mutation accumulation studies in evolutionary genetics. Pp. 151-172. Annual Review of Ecology Evolution and Systematics.

Massey, S. E. 2008. The Proteomic constraint and its role in molecular evolution. Mol. Biol. Evol. 25:2557-2565.
